# Some Pharmacodynamic Aspects of Cefepime

**DOI:** 10.1155/2013/381910

**Published:** 2012-11-07

**Authors:** Mossad Gamaleddin Ahmed Elsayed, Ashraf Abdelhakim Ahmed Elkomy, Mohamed Elbadawy

**Affiliations:** Pharmacology Department, Faculty of Veterinary Medicine, Benha University, P.O. Box 13736, Moshtohor, Toukh, Elqaliobiya, Egypt

## Abstract

Some pharmacodynamic effects of cefepime, a new injectable semisynthetic cephalosporin, were studied in laboratory animals and the following results were obtained. Cefepime maximally stimulated isolated guinea pig's ileum, rat's colon (80 *μ*g/mL bath), and rabbit's duodenum (400 *μ*g/mL bath). Contrarily, complete relaxation of isolated rat's fundic strip was produced by 80 *μ*g/mL bath. Effects of cefepime on isolated rat's uterine muscle were different according to stage of sex cycle. Cefepime did not induce any effects on the resting tonus of isolated guinea pig's tracheal chain and rabbit's aortic strip. Concentrations of 200 and 400 *μ*g/mL bath induced marked inhibition in the force of muscular twitches of the isolated frog's gastrocnemius muscle which was less potent than that induced by procaine hydrochloride 2%. Cefepime completely blocked the neuromuscular transmission of frog's rectus abdominis muscle (40 *μ*g/mL bath) and rat's phrenic nerve hemidiaphragm preparation (200 *μ*g/mL bath). This blockade was reversed by acetylcholine and neostigmine. Cefepime produced dose-dependent negative inotropic effect on isolated rabbit's heart and guinea pig's auricles. There were no changes in blood pressure and rate of respiration in anaesthetized dog after cefepime injection. These findings indicate that cefepime has a low potential to produce adverse reactions at therapeutic doses.

## 1. Introduction

Cefepime, a parenteral fourth generation cephalosporin antibiotic, is an established and generally well tolerated drug with a broad spectrum of antibacterial activity. Cefepime has *in vitro* activity against Gram positive and Gram negative organisms and is stable against many of the common plasmid and chromosome mediated beta lactamases [1–5].

Expanded information concerning the pharmacodynamic effects of cefepime will be of benefits to both physicians and their patients. Therefore, the purpose of this study was to investigate some pharmacodynamic effects of cefepime on smooth, skeletal, and cardiac muscles, as well as on systemic blood pressure, respiration, and electrocardiographic changes in guinea pigs, rabbits, rats, frogs, and dogs.

## 2. Materials and Methods

### 2.1. Materials

#### 2.1.1. Cefepime

 Cefepime is a new semisynthetic, broad spectrum, fourth generation cephalosporin antibiotic formulated for parenteral administration in strengths equivalent to 500 mg, 1 g, and 2 g of cefepime. It was produced by Bristol Myers Squibb Company (Egypt) and has the commercial name Maxipime.

#### 2.1.2. Laboratory Animals

Guinea pigs of both sexes and different weights (300–450 g) were used for investigating the effect of cefepime on the isolated ileum, auricles, and tracheal strips. Rabbits of both sexes and different weights (1500–2000 kg) were used for studying the effect of cefepime on isolated small intestines, heart, and electrocardiograph changes. Rats of both sexes and different weights (150–220 g) were used for studying the effects of cefepime on isolated colon, fundic strip, and uterine muscle in different stages of sex cycle and phrenic nerve hemidiaphragm. Egyptian toads were used for studying the effect of cefepime on isolated rectus abdominis muscle and sciatic nerve gastrocnemius muscle preparations. Mongrel dogs of both sexes weighing (15–20 kg) were employed for studying the effects of cefepime on blood pressure and rate of respiration. All animals were maintained on feed and water ad libitum and were kept in a good ventilation room at 25°C.

### 2.2. Methods

The method explained by Valeri et al. [6] was used for studying the effect of cefepime on the isolated ileum of guinea pigs. The method described elsewhere [7] was used for studying the effect of cefepime on isolated rabbit's duodenum, rat's colon, and uterine muscle of rats at various stages of sex cycle. The effect of cefepime on isolated rat's fundic strip was investigated according to the method described by Milenov and Kalfin [8]. The effect of cefepime on isolated guinea pig tracheal smooth muscle was studied using the glass jar bath apparatus [9]. The method described by Barlow et al. [10] was used for investigating the effect of cefepime on frog's gastrocnemius muscle-sciatic nerve preparation. The effect of cefepime on the isolated frog's rectus abdominis muscle was investigated [7]. The effect of cefepime on the isolated rat's phrenic nerve hemidiaphragm was studied by using the method described by Bulbring [11]. The glass jar bath was used for studying the effect of cefepime on isolated guinea pig's auricles [12]. The method using Gunn's apparatus (heart infusion assembly) was used for studying the effect of cefepime on rabbit's heart [13]. The effect of intravenous injection of cefepime on blood pressure and rate of respiration in an anaesthetized dog and the electrocardiographic changes in conscious rabbits after intramuscular injection of cefepime were performed using the method described by Jackson [14].

## 3. Results

**Figure 1:**
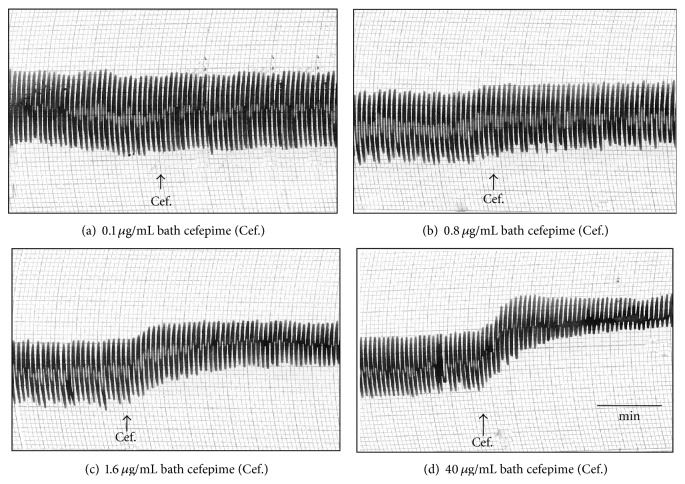
Effect of cefepime (Cef.) on isolated rabbit's duodenum.

**Figure 2:**
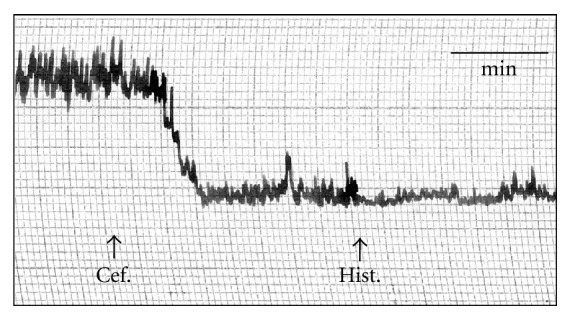
Site of action of cefepime (Cef.) on the isolated rat's fundic strip. 60 *μ*g/mL bath cefepime (Cef.) followed by histamine (Hist.) 2 *μ*g/mL bath.

**Figure 3:**
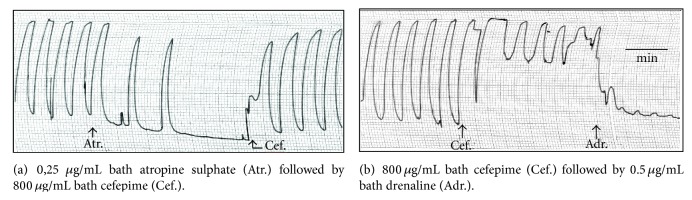
Site of action of cefepime (Cef.) on isolated rat's uterus during estrus stage.

**Figure 4:**
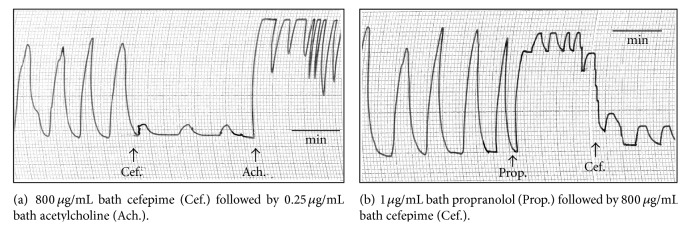
Site of action of cefepime (Cef.) on isolated rat's uterus during late pregnant stages.

**Figure 5:**
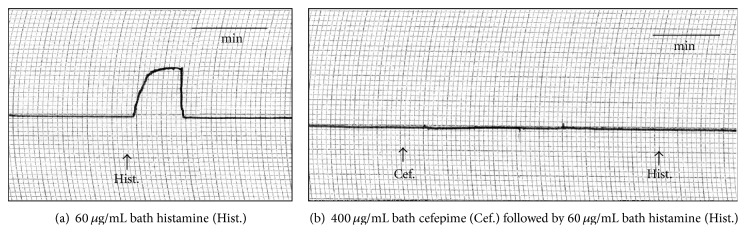
Site of action of cefepime (Cef.) on isolated guinea pig's tracheal chain.

The effect of cefepime on isolated guinea pig's ileum, rabbit's duodenum, rat's colon, and rat's fundic strip and uterine motility of female rats at various stages of sex cycle have been recorded and presented in Table 1. Action of cefepime on the rabbit duodenal motility is shown in Figure 1. The rat's fundic strip was depressed by cefepime in a dose-dependent manner and histamine was not able to produce its stimulant effect in presence of cefepime (Figure 2) so cefepime might appear to have an antihistaminic-like effect. Cefepime stimulated the uterine motility during estrus and nonestrus stages and depressed it during early and late pregnancy and these findings might be attributed to the direct effect of cefepime (Figures 3(a) and 3(b) and Figures 4(a) and 4(b)). Cefepime had no effect on the resting tonus of the isolated guinea pig's tracheal chain but histamine (60 *μ*g/mL bath) was not able to produce its contractile effect in the presence of cefepime (400 *μ*g/mL bath) as shown in Figure 5. This indicated that cefepime might have an antihistaminic like effect on this tissue.

**Figure 6:**
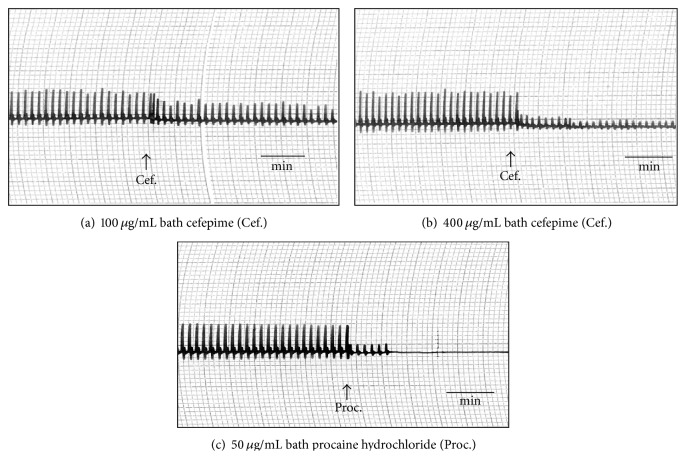
Effect of cefepime (Cef.) on isolated frog's gastrocnemius muscle.

**Figure 7:**
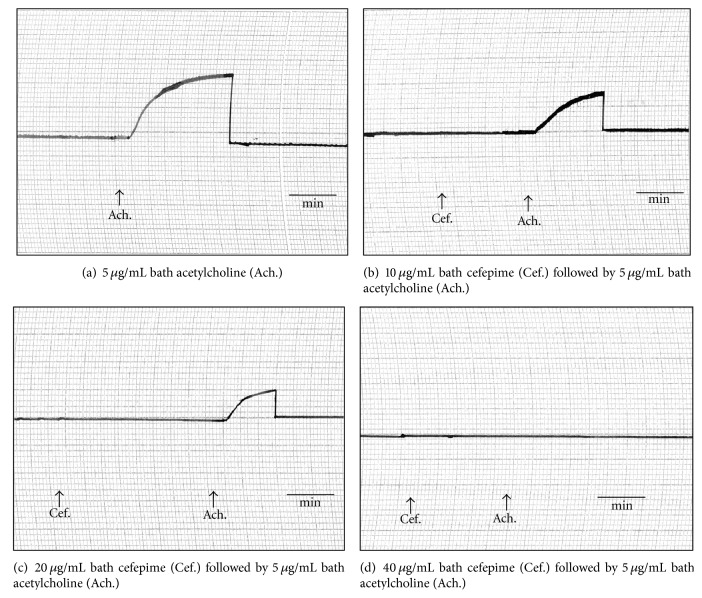
Effect of cefepime (Cef.) on isolated frog's rectus abdominis muscle.

**Figure 8:**
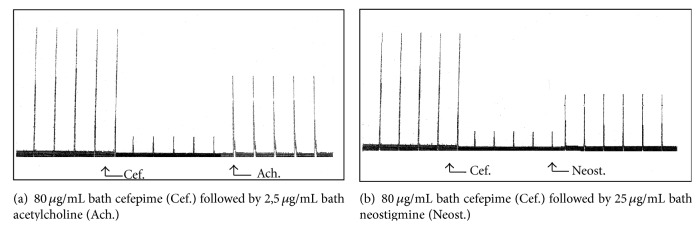
Site of action of cefepime (Cef.) on isolated rat's phrenic nerve hemidiaphragm.

Cefepime at concentrations of 200 and 400 *μ*g/mL bath induced marked inhibition in the force of muscular twitches of frog's gastrocnemius muscle which was less potent than that induced by procaine hydrochloride 2% (Figure 6). Concentrations of 20 *μ*g/mL bath caused marked decrease in the contracture of frog's rectus abdominis muscles while complete blockade was produced in the presence of 40 *μ*g/mL bath of cefepime (Figure 7). The concentrations of 80 *μ*g/mL bath produced marked inhibition in the force of rat's phrenic nerve hemidiaphragm which was reversed by 2.5 *μ*g acetylcholine/mL bath (Figure 8(a)) or 25 *μ*g neostigmine/mL bath (Figure 8(b)). This indicated that cefepime might act directly to induce neuromuscular blockade.

**Figure 9:**
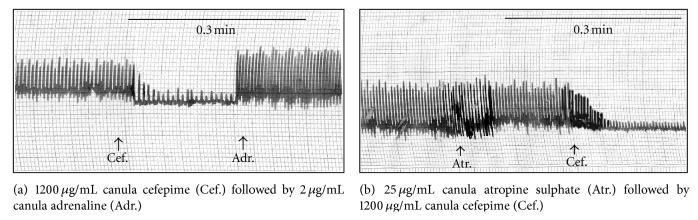
Site of action of cefepime (Cef.) on isolated rabbit's heart.

 The effect of gradually increased concentrations of cefepime on isolated guinea pig's auricles, rabbit's heart, and aortic strip has been demonstrated in Table 2. Cefepime depressed the isolated perfused rabbit's heart and this depression might be attributed to the direct effect of cefepime (Figure 9).

**Figure 10:**
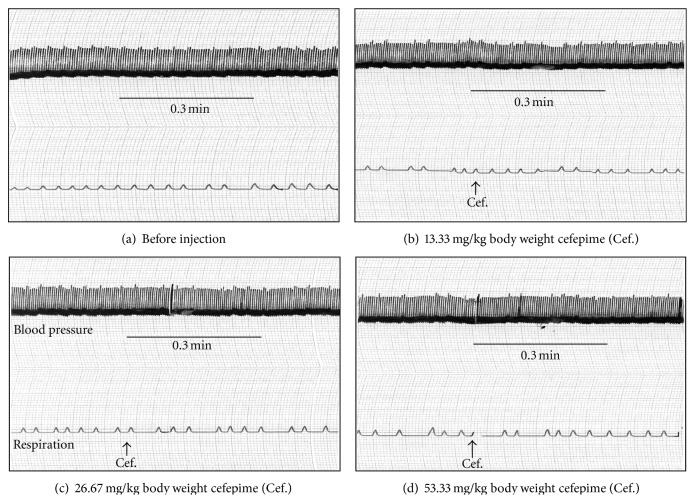
Effect of cefepime (Cef.) on blood pressure and rate of respiration in anesthetized dogs.

Intravenous injection of cefepime in doses of 13.33, 26.67, and 53.33 mg/kg body weight had no effect on blood pressure and rate of respiration in anesthetized dogs (Figure 10). Single intramuscular injection of 23.33, 46.67, and 93.33 mg/kg body weight induced no effects on the ECG parameters along period of 8 hours in conscious rabbits as shown in Table 3.

## 4. Discussion

### 4.1. Effect of Cefepime on Smooth Muscle Preparations

The present investigation showed that cefepime *in vitro *stimulated the contractility of guinea pig's ileum, rat's colon, and rabbit's duodenum. The stimulatory effect of cefepime was proportional to the graded tested concentrations. Presence of atropine sulphate as a muscarinic cholinergic receptor blocker and large dose of nicotine sulphate as ganglionic (nicotinic receptor) blocker did not inhibit the stimulatory effect of cefepime. In addition, the adrenaline as an adrenoceptor agonist produced its inhibitory effect in presence of cefepime. These results proved that; the cefepime might directly stimulate the intestinal smooth muscles of rabbit's duodenum, guinea pig's ileum, and rat's colon. These obtained results were similar to the fact that cefeperazone *in vivo* enhanced the ileal motility in guinea pigs at 62.5 and 125 mg/kg, respectively, and promoted gastrointestinal propulsion of barium sulfate meal in mice at 1000 mg/kg and* in vitro* enhanced slightly the motility of isolated rabbit's gastrointestinal tract at 0.001 g/mL [15]. Also these findings were similar to the fact that the spontaneous motility of smooth muscle *in situ* was temporarily increased with 800 mg/kg cefminox when administered intravenously and in upper doses [16, 17]. In contrast, cefadroxil had no effects on the isolated smooth muscle organs and the passage of charcoal meal in mice [18]. In addition, ceftizoxime sodium neither affected the spontaneous motility of isolated rabbit's and guinea pig's ileum at concentration equal to 10^−2^ g/mL nor interacted with acetylcholine or histamine on the isolated guinea-pig's preparation [19]. The spontaneous movement and tone of isolated ileum, colon, and acetylcholine-, histamine-, nicotine-, or barium chloride-induced contraction of ileum were not affected following cefbuperazone application [20]. Furthermore, cefprozil did not affect the isolated smooth muscles of rat's uterus, guinea pig's ileum, or rabbit's duodenum and did not influence ganglionic transmission in cats [21]. Cefepime had no effect on the intestinal smooth muscle and did not show any antagonism against some smooth muscle contracting drugs [22]. Cefamandole at concentrations of 512 and 1024 micrograms/mL bath caused complete relaxation in isolated guinea pig's ileum and rabbit's duodenum, respectively [23]. The maximum contractile responses to carbachol and histamine were significantly reduced in response to the ceftriaxone sodium [24]. 

Cefepime inhibited the contractility of the rat's fundic strip. This inhibitory effect was dose dependent. In presence of cefepime, histamine was unable to stimulate the fundic strip. This result indicated that cefepime had an antihistaminic like effect on the rat's fundic strip. The obtained results came in harmony with the dose-dependently suppressed effect of ceftizoxime sodium after intravenous dose of 320 to 1000 mg/kg of the spontaneous contraction of the pyloric antrum in morphine-urethane-anesthetized dogs [19], while Cefadroxil had no effects on the motility of the stomach *in situ* in rabbits [18]. On the other hand, cefotaxime, ceftriaxone, and ceftazidime produced concentration-dependent tonic contractions of rat's fundus [25] and cefamandole had stimulatory effect on the rat's fundic strips [23].

Cefepime stimulated the uterine motility during estrus and nonestrus and inhibited the uterine motility during early and late pregnant stage. The effect was dose dependent. These effects might be attributed to the direct action of the cefepime on the isolated uterus. During estrus and nonestrus, and in presence of atropine sulphate (0.25 *μ*g/mL bath), cefepime induced its stimulant effect and the adrenaline (0.5 *μ*g/mL bath) relaxed the uterus after its stimulation with 800 *μ*g/mL bath cefepime. During the early and late pregnant stages, the addition of acetylcholine in a small concentration (0.25 *μ*g/mL bath) produced its stimulatory effect in the presence of cefepime (800 *μ*g/mL bath) and the cefepime in the same concentration relaxed the uterus after its stimulation with 1 *μ*g propranolol/mL bath. The obtained results were consistent with the uterine stimulant effect of cefeperazone during estrus and nonestrus stages in four of six experiments and the uterine depressant effect of it in two of six experiments while during pregnancy, cefoperazone might not affected or depressed and/or stimulated the uterine motility [15]. Also the present results were similar to the effect of cefteram pivoxil during nonpregnancy. Cefteram pivoxil increased both the force and frequency of the uterine motility in four of six experiments and had no changes in four of another six experiments. During pregnancy it induced no changes in four of six experiments and increased in four of another six experiments [26]. The obtained results during estrus and nonestrus stages were agreeable also with the effect of cefamandole on uterine contractility; cefamandole concentrations of 2048 and 4096 micrograms cefamandole/mL bath caused marked stimulation in force and frequency of rat uterine muscle in all stages of sex cycle [23]. In another observation, cefepime had no effect on the delivery status of the offspring rats [27], and the spontaneous movement and tone of isolated uterus were not affected following cefbuperazone application [20]. Effects of beta-lactam antibiotics on smooth muscle isolated preparations were tissue and species dependent, indicating selectivity of their action [25].

The guinea pig's tracheal smooth muscles seemed to be insensitive to the tested concentrations of cefepime. In presence of cefepime, histamine was not able to produce its stimulatory effect, thus cefepime blocked the action of histamine on the tracheal smooth muscles. The obtained results in this study were similar to the effect of cefprozil and cefamandole which had no effect on the tracheal smooth muscles in different graded concentrations. The two antibiotics blocked the stimulatory effect of histamine on the guinea pig's tracheal muscles in a dose-dependent manner [21, 23]. On the other hand, ceftizoxime and cefminox relaxed the resting tonus of the isolated guinea pig's tracheal chain preparation at a concentration of 10^−3^ g/mL [16, 19]. Cefoperazone and cefteram pivoxil caused slight stimulation of the isolated guinea pig's tracheal smooth muscles in a concentration as high as 10^−2^ g/mL and 10^−3^ g/mL, respectively [15, 26]. These results might be attributed to the high concentration of the used antibiotics than those used in this study.

### 4.2. Effect of Cefepime on the Skeletal Muscle Preparations

The effect of cefepime on skeletal muscle preparations (frog's gastrocnemius muscle sciatic nerve, frog's rectus abdominis muscle, and rat's phrenic nerve hemidiaphragm) was investigated. The cefepime elicited a marked neuromuscular blocking activity in response to indirect muscle twitches; also cefepime exhibited a local anaesthetic like activity on frog's gastrocnemius sciatic nerve preparation. 

Trials were performed to detect the site of action of cefepime on the skeletal muscle preparations. The results showed that cefepime did not impair the stimulatory effect of neostigmine and acetylcholine on rat's phrenic nerve hemidiaphragm preparation. Therefore, the neuromuscular blocking effect of cefepime seemed to be attributed to two mechanisms; the first might be due to local anaesthetic effect of cefepime which is responsible for blocking of conduction through sciatic and phrenic nerve. The second mechanism might be attributed to calcium ions antagonistic effect of the cefepime. Calcium ions influx is necessary for acetylcholine release as well as other neurotransmitters and hormones [28].

The neuromuscular blocking activity of cefepime on skeletal muscle preparations in the present work was similar to the fact that the twitch tension of gastrocnemius muscle evoked by electrical stimulation of sciatic nerve was slightly reduced following administraton of cefminox and cefteram pivoxil, respectively [16, 26]. Also the present effect was similar to the fact that cefepime reduced spontaneous locomotor activity in mice [22]. Cefamandole had a neuromuscular blocking effect on isolated frog's gastrocnemius muscle, frog's rectus abdominis muscle, and rat's phrenic nerve hemidiaphragm [23].

The obtained results were inconsistent with the fact that ceftizoxime did not affect the contractile response of the isolated rat's diaphragm to electrical stimulation of the phrenic nerve at concentration of 10^−3^ g/mL [19]. Cefoperazone enhanced slightly the twitch tension of musculus gastrocnemius induced by electrical stimulation in rats at 500  mg/kg body weight [15]. Cefbuperazone had no effect on the neuromuscular junction [20].

From the present study it could be concluded that cefepime has depressant effect on the skeletal muscles in a manner similar to that of the local anesthetic effect of procaine hydrochloride.

### 4.3. Effect of Cefepime on the Cardiovascular Muscle Preparations

The obtained results in this study on the cardiovascular muscles proved that cefepime had a negative inotropic effect on the isolated guinea pig's auricles and rabbit's heart. Cefepime produced a direct and dose dependent depression of the myocardial contractility. This negative inotropic effect of cefepime was not referred to either ß1 adrenergic blocking effect or cholinergic stimulant effect, as adrenaline (2 *μ*g/mL canula) was able to produce its cardiac stimulatory effect in presence of cefepime (1200 *μ*g/mL canula) and after addition of atropine sulphate (25 *μ*g/mL canula), cefepime (1200 *μ*g/mL canula) was able to produce its inhibitory effect. 

Contraction of the cardiac cells is believed to be dependent upon the intracellular concentration of available calcium ions in the vicinity of the contractile apparatus [29] so the direct myocardial depressant effect of cefepime in the present work might be attributed to a modification of calcium function.

The negative inotropic effect of cefepime on guinea pig's auricles and rabbit's heart in the present work was similar to the direct depressant effect of cefamandole on the contractility of isolated guinea pig's auricles and rabbit's heart in a dose-dependent manner [23]. This obtained result was not consistent with that; cefadroxil and ceftizoxime sodium in a dose of 10^−2^ g/mL had no effects on the isolated hearts of rabbits and the spontaneous movement in isolated guinea pig's atrium, respectively [18, 19]. The obtained results were also inconsistent with the fact that cefminox did not affect the spontaneous contraction of isolated guinea pig's atria and the blood vessels in perfused rabbit's ears [30]. 

It was observed that cefepime had no effect on the smooth muscle of aorta. In the presence of cefepime, noradrenaline was not able to produce its stimulatory effect, thus cefepime appeared to cause an alpha adrenergic blocking like effect on isolated rabbit's aortic strip. This result was consistent with the fact that cefbuperazone, cefteram pivoxil, and cefamandole, respectively, did not affect the rabbit's descending aorta and the adrenaline and noradrenaline fail to produce its stimulatory effect in the presence of these antibiotics [20, 23, 26]. This was inconsistent with the fact that cefoperazone potentiated the presser response to adrenaline in dogs [15]. 

### 4.4. Effect of Cefepime on Blood Pressure and Respiration

After intravenous injection of cefepime in doses of 13.33, 26.67, and 53.33 mg/kg body weight, no changes were induced in blood pressure and rate of respiration in an anaesthetized dog over a period of half hour after each dose. This is supported by the lack of cefepime effects on the electrocardiographic parameters in this study. The depressant effects of cefepime on isolated heart and auricles and the alpha adrenoceptor blocking effects of cefepime on the isolated aortic strip in this study might be attributed to the higher doses of cefepime in the organ bath. The obtained results were consistent with the fact that cefadroxil induced no marked changes in the respiration and blood pressure in anesthetized dogs [18]. 

The obtained findings were inconsistent with the fact that cefoperazone increased transiently the respiratory rate and potentiated the depressor response to acetyl choline at 125 mg/kg, increased femoral blood flow, and potentiated the presser response to adrenaline in dogs [15]. Also, cefbuperazone caused transient increase of respiratory rate, slight hypotension, and transient increase of femoral blood flow in dogs at intravenous doses of 250–1000 mg/kg [20]. Moreover, cefminox slightly raised a level of blood pressure in dogs when intravenously given more than 400 mg/kg [30]. Cefteram caused a slight hypotension and increased both respiratory rate and femoral blood flow [26]. The respiration and blood pressure were depressed by cefepime [22]. In the same direction, cefamandole in a dose of 53.2 mg/kg body weight in anaesthetized dogs caused very marked hypotensive effects and decrease in rate of respiration [23]. These might be attributed to the higher doses used in these studies. 

### 4.5. Effect of Cefepime on Electrocardiographic Changes

After single intramuscular injection of the 23.33, 46.66, and 93, 32 mg/kg body weight of cefepime in conscious rabbits, no changes were observed in electrocardiographic parameters during a period of 8 hours after injection. This result was consistent with those observed in rabbits [15, 20, 21] and dogs [26]. This result was inconsistent with the fact that the electrocardiogram was affected by cefepime and this might be attributed to the use of higher doses (1000 mg/kg body weight) of cefepime, a factor that might have caused modification of calcium ions function [22].

## 5. Conclusion

From the present study it could be noticed that cefepime stimulates the smooth muscles of intestines and uterus during estrus and nonestrus and depresses those of stomach, uterus during early and late pregnancy as well as cardiac muscles. Cefepime also has depressant effect on the skeletal muscles in a manner similar to that of procaine hydrochloride. The study also showed that cefepime has no effect on blood pressure, respiration, and ECG. From the present results and previous reports, it is evident that cefepime has strong potential as highly safe and useful antibiotic agent in clinical use. 

## Figures and Tables

**Table 1:** The effect of cefepime on isolated guinea pig's ileum, rabbit's duodenum, rat's colon, rat's fundic strip, and uterine motility of female rats.

Concentrations (*μ*g/mL bath)	Responses of							
	Guinea pig's ileum	Rabbit's duodenum	Rat's colon	Rat's fundic strip	Rat's uterine muscle			
					Non estrus	Estrus	Early pregnant	Late pregnant
0.0125	No effect	No effect	No effect	No effect	No effect	No effect	No effect	No effect
5	Slight stimulation	Slight stimulation	Slight stimulation in the force	Slight inhibition in the force	No effect	No effect	No effect	No effect
10	Marked stimulation	Slight stimulation	Marked stimulation in force and frequency	Marked inhibition in the force and rate of contraction	No effect	No effect	No effect	No effect
20	Marked stimulation	Slight stimulation	Marked stimulation in force and frequency	Marked inhibition in the force and rate of contraction	No effect	No effect	No effect	No effect
40	Marked stimulation	Marked stimulation	Marked stimulation in force and frequency	Marked inhibition in the force and rate of contraction	No effect	No effect	Slight inhibition in the force and frequency	Slight inhibition in the force
60	Marked stimulation	Marked stimulation	Marked stimulation in force and frequency	Marked inhibition in the force and rate of contraction	Increase in frequency	Increase in frequency	Slight inhibition in the force and frequency	Slight inhibition in the force and frequency
80	Maximum stimulation	Marked stimulation	Maximum stimulation	Complete inhibition	Increase in frequency	Increase in frequency	Slight inhibition in the force and frequency	Slight inhibition in the force and frequency
400	—	Maximum stimulation	—	—	Marked increase in frequency	Marked increase in force and frequency	Marked inhibition in force and frequency	Marked inhibition in force and frequency
800	—	—	—	—	Maximum stimulation	Maximum stimulation	Complete relaxation	Complete relaxation

(—) not done.

**Table 2:** The effect of cefepime on isolated guinea pig's auricles, rabbit's heart, and rabbit's aortic strip.

Concentrations (*μ*g/mL bath)	Responses of		
	Guinea pig's auricles	Rabbit's heart	Rabbit's aortic strip
0.0125	No effect	No effect	No effect
5	No effect	No effect	No effect
10	No effect	No effect	No effect
20	No effect	No effect	No effect
40	No effect	No effect	No effect
60	No effect	No effect	No effect
80	No effect	No effect	No effect
100	No effect	Slight negative inotropic effect.	No effect
200	Slight negative inotropic effect.	Slight negative inotropic effect.	No effect
400	Slight negative inotropic effect.	Slight negative inotropic effect.	No effect
800	Marked negative inotropic effect	Marked negative inotropic effect	No effect

**Table 3:** Effect of single intramuscular injection of 93.33 mg/kg body weight of cefepime on electrocardiographic changes in conscious rabbits (*n* = 3).

ECG parameters	Time (hours)						
	0	0.25	0.5	1	2	4	8
P-wave:							
(a) Amplitude (m·v.)	0.15 ± 0.0	0.15 ± 0.0	0.15 ± 0.0	0.15 ± 0.0	0.15 ± 0.0	0.15 ± 0.0	0.15 ± 0.0
(b) Duration (m·sec.)	38.66 ± 0.67	34 ± 0.58	34 ± 1.15	37.66 ± 1.45	36.33 ± 0.88	37.67 ± 0.33	40 ± 0.0
*PR-interval:* (m·sec.)	20 ± 0.0	20 ± 0.0	20 ± 0.0	20 ± 0.0	20 ± 0.0	20 ± 0.0	20 ± 0.0
QRS-complex:							
(a) Amplitude (m·v.)	0.53 ± 0.033	0.43 ± 0.02	0.53 ± 0.017	0.53 ± 0.033	0.57 ± 0.033	0.43 ± 0.02	0.55 ± 0.003
(b) Duration (m·sec.)	20 ± 0.0	20 ± 0.0	20 ± 0.0	20 ± 0.0	20 ± 0.0	20 ± 0.0	20 ± 0.0
*ST-segment: *(m·sec.)	40 ± 0.0	40 ± 0.0	40 ± 0.0	40 ± 0.0	40 ± 0.0	40 ± 0.0	40 ± 0.0
T-wave							
(a) Amplitude (m·v.)	0.19 ± 0.007	0.16 ± 0.007	0.15 ± 0.0	0.19 ± 0.007	0.20 ± 0.0	0.19 ± 0.007	0.20 ± 0.0
(b) Duration (m·sec.)	40 ± 0.0	42 ± 1	42 ± 1	39.33 ± 0.67	40 ± 0.0	40.67 ± 0.67	40 ± 0.0
*QT-interval:* (m·sec.)	140 ± 1.16	136.3 ± 0.88	135 ± 0.0	138 ± 1.15	140.67 ± 0.67	142.3 ± 1.45	142.3 ± 1.45
*H.R:* (beets/min)	300 ± 0.0	300 ± 0.0	300 ± 0.0	300 ± 0.0	300 ± 0.0	300 ± 0.0	300 ± 0.0

## References

[1] Sanders C. C. (1993). Cefepime: the next generation?. *Clinical Infectious Diseases*.

[2] Chapman T. M., Perry C. M. (2003). Cefepime: a review of its use in the management of hospitalized patients with pneumonia. *American Journal of Respiratory Medicine*.

[3] Huang S. S., Lee S. C., Lee N., See L. C., Tsai M. H., Shieh W. B. (2007). Comparison of *in vitro* activities of levofloxacin, ciprofloxacin, ceftazidime, cefepime, imipenem, and piperacillin-tazobactam against aerobic bacterial pathogens from patients with nosocomial infections. *Journal of Microbiology, Immunology and Infection*.

[4] Yahav D., Paul M., Fraser A., Sarid N., Leibovici L. (2007). Efficacy and safety of cefepime: a systematic review and meta-analysis. *Lancet Infectious Diseases*.

[5] Crandon J. L., Kuti J. L., Jones R. N., Nicolau D. P. (2009). Comparison of 2002-2006 OPTAMA programs for US hospitals: focus on gram-negative resistance. *Annals of Pharmacotherapy*.

[6] Valeri P., Martinelli B., Morrone L. A., Severini C. (1990). Reproducible withdrawal contractions of isolated guinea-pig ileum after brief morphine exposure: effects of clonidine and nifedipine. *Journal of Pharmacy and Pharmacology*.

[7] Members of the Departement of Pharmacology, University of Edinburgh (1968). *Pharmacological Experiments on Isolated Preparation*.

[8] Milenov K., Kalfin R. (1996). Cholinergic-nitrergic interactions in the guinea-pig gastric fundus. *Neuropeptides*.

[9] Schlemper V., Calixto J. B. (1995). Mechanisms involved in the relaxant response of bradykinin in epithelium intact strips of the guinea-pig trachea. *European Journal of Pharmacology*.

[10] Barlow R. B., Crawford T. B. B., Perry W. L. M. (1974). *Pharmacological Experiments on Isolated Preparations*.

[11] Bulbring E. (1946). Observation on the isolated rat phrenic nerve diaphragm preparation. *British Journal of Pharmacology and Chemotherapy*.

[12] Vasconcelos C. M. L., Araújo M. S., Silva B. A., Conde-Garcia E. A. (2005). Negative inotropic and chronotropic effects on the guinea pig atrium of extracts obtained from Averrhoa carambola L. leaves. *Brazilian Journal of Medical and Biological Research*.

[13] Hondeghem L. M., Hoffmann P. (2003). Blinded test in isolated female rabbit heart reliably identifies action potential duration prolongation and proarrhythmic drugs: importance of triangulation, reverse use dependence, and instability. *Journal of Cardiovascular Pharmacology*.

[14] Jackson D. E. (1939). *Experimental Pharmacology and Materia Media*.

[15] Takai A., Hirai S., Watanabe I. (1980). General pharmacology of cefoperazone, a new cephalosporin antibiotic. *Japanese Journal of Antibiotics*.

[16] Yamaki Y., Shibazaki Y., Kadosaka H. (1984). Pharmacological studies on a new cephamycin, MT-141. (2) Its effect on preparations of neuromuscular junction, smooth muscle organs and gastro-intestinal system. *Japanese Journal of Antibiotics*.

[17] Elsayed M. G., Elkomy A. A., Aboubakr M. H. (2011). Effect of ceftriaxone on isolated smooth, cardiac muscles and neuromuscular junctions. *International Journal for Agro Veterinary and Medical Sciences*.

[18] Hasegawa Y., Muto N., Morita M. (1979). General pharmacology of cefadroxil. *Japanese Journal of Antibiotics*.

[19] Honda F., Ono T., Itoh N. (1980). General pharmacology of ceftizoxime sodium. *Drug Research*.

[20] Takai A., Hirai S., Watanabe I. (1982). General pharmacology of T-1982, a new cephamycin antibiotic. *Japanese Journal of Antibiotics*.

[21] Goto A., Amano M., Sakai A., Hara M., Takahashi N. (1990). General pharmacology of cefprozil. *Japanese Journal of Antibiotics*.

[22] Goto A., Amano M., Sakai A., Hara M., Takahashi N. (1992). General pharmacology of cefepime. *Japanese Journal of Antibiotics*.

[23] El-Sayed M. G. A., Hassanin M. R., Hafez M. H., El-Komy A. A. A., Mohamed A. (1997). Some pharmacodynamic and biochemical aspects of cefamandole. *Deutsche Tierarztliche Wochenschrift*.

[24] Ceran C., Karadas B., Kaya T., Arpacik M., Bagcivan I., Sarac B. (2006). Do antibiotics contribute to postoperative ileus? Contractile responses of ileum smooth muscle in guinea pigs to long-term parenteral ceftriaxone and ampicillin. *ANZ Journal of Surgery*.

[25] Janković S. M., Kouvelas D., Mitrović M. (1996). Spasmogenic action of beta-lactam antibiotics an the gastrointestinal tract of experimental animals. *Indian Journal of Medical Research*.

[26] Hirai S., Kodama T., Hiraiwa T. (1986). General pharmacology of cefteram pivoxil, a new oral cephem antibiotic. *Japanese Journal of Antibiotics*.

[27] Kai S., Kohmura H., Ishikawa K. (1992). Reproductive and developmental toxicity studies on cefepime dihydrochloride adminstered subcutaneously to rats during the premating, gestation and lactation periods. *Japanese Journal of Antibiotics*.

[28] Rubin R. P. (1970). The role of calcium in the release of neurotransmitter substances and hormones. *Pharmacological Reviews*.

[29] Katz A. M., Repke D. I. (1973). Calcium-membrane interactions in the myocardium: effects of ouabain, epinephrine and 3′, 5′-cyclic adenosine monophosphate. *The American Journal of Cardiology*.

[30] Kurebe M., Asaoka H., Yamaki Y. (1984). Pharmacological studies on a new cephamycin, MT-141. (1) Its effect on central nervous system, respiration and cardiovascular system. *Japanese Journal of Antibiotics*.

